# Protein sequence database for pathogenic arenaviruses

**DOI:** 10.1186/1745-7580-3-1

**Published:** 2007-02-08

**Authors:** Huynh-Hoa Bui, Jason Botten, Nicolas Fusseder, Valerie Pasquetto, Bianca Mothe, Michael J Buchmeier, Alessandro Sette

**Affiliations:** 1 La Jolla Institute for Allergy and Immunology, Division of Vaccine Discovery, 9420 Athena Circle, La Jolla, CA 92037, USA; 2 The Scripps Research Institute, Molecular & Integrative Neurosciences Department, 10550 North Torrey Pines Road, La Jolla, CA 92037, USA; 3 California State University, Department of Biology, San Marcos, CA 92096, USA

## Abstract

**Background:**

Arenaviruses are a family of rodent-borne viruses that cause several hemorrhagic fevers. These diseases can be devastating and are often lethal. Herein, to aid in the design and development of diagnostics, treatments and vaccines for arenavirus infections, we have developed a database containing protein sequences from the seven pathogenic arenaviruses (Junin, Guanarito, Sabia, Machupo, Whitewater Arroyo, Lassa and LCMV).

**Results:**

The database currently contains a non-redundant set of 333 protein sequences which were manually annotated. All entries were linked to NCBI and cited PubMed references. The database has a convenient query interface including BLAST search. Sequence variability analyses were also performed and the results are hosted in the database.

**Conclusion:**

The database is available at  and can be used to aid in studies that require proteomic information from pathogenic arenaviruses.

## Background

Arenaviridae are a family of viruses whose members are associated with rodent-transmitted disease in humans. Each virus usually is associated with a particular rodent host species in which it is maintained. Arenavirus infections, occur when an individual comes into contact with the excretions of an infected rodent, are relatively common in humans in some area of the world and primarily cause hemorrhagic fevers, including Lassa fever (LF; Lassa virus), Argentine hemorrhagic fever (AHF; Junin virus), Bolivian hemorrhagic fever (BHF; Machupo virus), Venezuelan hemorrhagic fever (VHF; Guanarito virus) and Brazillian hemorrhagic fever (BrHF; Sabia virus) [1-6]. These diseases can be devastating and often lethal. Lymphocytic choriomeningitis virus (LCMV), a known human teratogen, can cause aseptic meningitis [7-9], and Whitewater Arroyo Virus (WWA) was recently attributed to two deaths in California [10,11].

The arenaviruses can be classified phylogenetically into Old World (which includes LCMV and Lassa virus) and New World; this latter group has been further divided into three lineages, A-C [12,13]. Except for WWA virus which belongs to lineage A, the four most pathogenic New World agents (Junin, Machupo, Guanarito and Sabia viruses) all belong to lineage B, suggesting that the highly pathogenic phenotype may derive from a common ancestral virus [12,14]. All of these viruses cause significant morbidity and mortality. Lassa virus and other hemorrhagic fever arenaviruses (Junin, Machupo, Guanarito and Sabia) are included in category A of potential bioterrorism microbial weapons [15].

Currently, there are no virus-specific treatments approved for use against arenavirus hemorrhagic fevers. Ribavirin is the only compound that has shown partial efficacy against some arenavirus infections [16] (successful against human Lassa infections only if given within the first week following disease onset [17]), and to date only one vaccine (against AHF) has been evaluated in humans [2]. Because of its severe morbidity and high mortality together with lack of immunization or effective treatment, scientists and researchers are challenged with developing containment, treatment, and vaccine strategies for arenavirus infection. For the purpose of developing diagnostic reagents and designing novel vaccine constructs, our group has been conducting active studies in identifying MHC class I and II restricted T cell epitopes from pathogenic arenaviruses. As a component of the studies, we have compiled and developed a database of protein sequences for the seven arenaviruses (Lassa, LCMV, Junin, Guanarito, Sabia, Machupo and WWA) known to cause disease in humans. Herein, we make this database available as a public resource to aid in studies that require proteomic information from pathogenic arenaviruses.

## Construction and content

Arenaviridae are enveloped viruses with a genome consisting of two single-stranded RNA, the small (S) and the large (L), segments. Each segment encodes two different proteins. The S RNA encodes the nucleocapsid protein (NP) and the glycoprotein precursor (GPC) which undergoes post-translational processing to yield two mature proteins (GP1 and GP2) [18]. The L RNA encodes the viral RNA-dependent RNA polymerase (L) and a zinc-binding matrix protein (Z) [19,20]. These four proteins (GPC, L, NP and Z) are the collection targets of our database.

**Figure 1:**
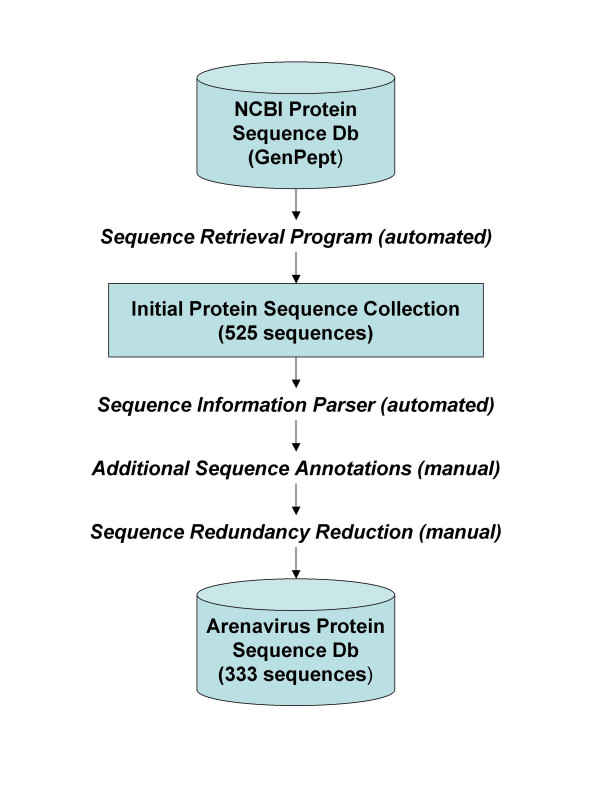
Arenavirus protein sequence database construction flowchart.

The process of compiling arenaviral protein sequences for the database includes 1) retrieving published sequences from NCBI, 2) parsing for sequence information, 3) manually verifying the information and performing additional annotations, and 4) removing duplicated entries. A schematic flow chart of this process is shown in Figure 1. MySQL was used as the storage database engine, Tomcat as the webserver, and Java servlets were used to develop the web interface.

To obtain protein sequences encoded by the seven pathogenic arenaviruses, we first searched the NCBI database through the use of an automated computer program developed in our laboratory. This program was written to: (1) search for protein sequence IDs, (2) retrieve protein sequence records and (3) parse the records into annotated fields. First, protein sequence IDs (GI numbers) were retrieved via an NCBI Esearch programming utility using NCBI taxonomy IDs as search parameters. Next, GI numbers were used to retrieve protein sequence records in the GenPept format. "NCBISequenceDB" java class from biojava 1.4 package was used to programmatically retrieve the GenPept sequence records. Finally, a customized java class was written to parse each record into annotated fields which include protein sequence data, source references, virus, strain and gene name.

Using the automated program, 525 protein sequences were retrieved from NCBI. Next, verification of sequence authenticity was performed via supporting publications and/or written summaries. Finally, we conducted manual protein sequence alignments to identify and remove duplications. Of the 525 protein sequences from the initial cohort, a total of 333 unique protein sequences from one or more strains of the seven pathogenic arenavirus viruses were obtained (Table 1). At present, the Z protein sequence has not been published for WWA virus. As a result of renewed interests in arenaviruses, we anticipate that more protein sequences will become available in the near future. We plan to periodically monitor the scientific literature and the NCBI database for new sequence depositions, and update our database accordingly.

**Table 1:** Arenavirus protein sequence distribution

	**Protein**				
		
**Virus**	**GPC**	**L**	**NP**	**Z**	***Total***
Guanarito	1	1	32	1	***35***
Junin	43	3	45	2	***93***
Lassa	12	7	64	6	***89***
LCMV	10	9	6	5	***30***
Machupo	28	4	30	3	***65***
Sabia	1	1	1	1	***4***
Whitewater Arroyo	2	1	14	0	***17***

***Total***	***97***	***26***	***192***	***18***	***333***

## Utility and discussion

### Arenavirus protein sequence annotation

To maximize the usefulness of the arenavirus protein sequence database to the scientific community, each record was annotated with specific information, including the host and geographical region from which each protein sequence was isolated and the passage history of each viral strains between its original isolation from its natural host and the time it was sequenced. Inclusion of the host that each protein sequence was isolated from is of potential relevance in studies examining specific host-derived immune pressure or host-specific viral adaptations. The inclusion, if available, of the geographical region is relevant in determining whether the available viral strains are represented in endemic locations. Finally, the passage history of each strain is relevant in the context of the high mutation rates associated with these RNA viruses and the potential for genetic changes to accumulate as a result of *in vitro *passage. Mutations generated as a result of viral passage in non-reservoir animals or cell lines would not be representative of the natural variation present in field or clinical strains. All annotated information was obtained via collected publications and/or via direct correspondence with the authors of a given protein sequence. Most protein sequences were derived from human infections, while the remaining samples came from naturally infected reservoir rodents. Universally, each of the viruses sequenced prior to 2002 was propagated in Vero E6 or BHK cell lines prior to sequencing of the viral genome.

### Search Interface

**Figure 2:**
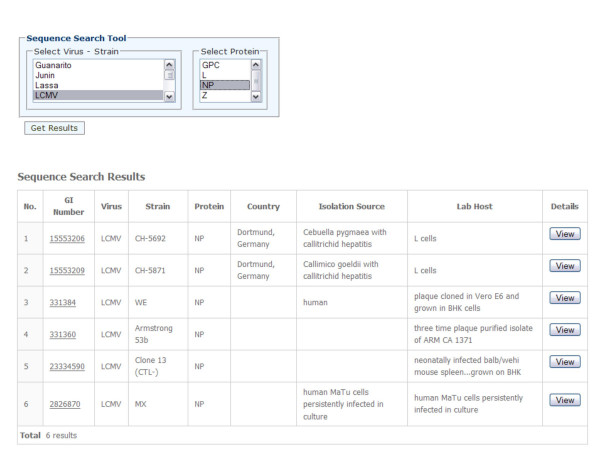
Sequence search interface.

**Figure 3:**
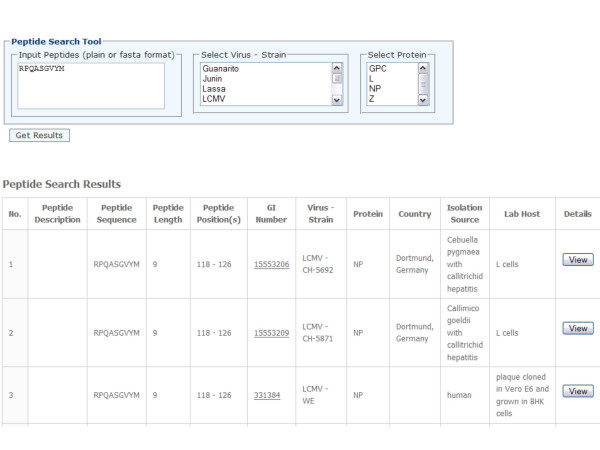
Peptide search interface.

**Figure 4:**
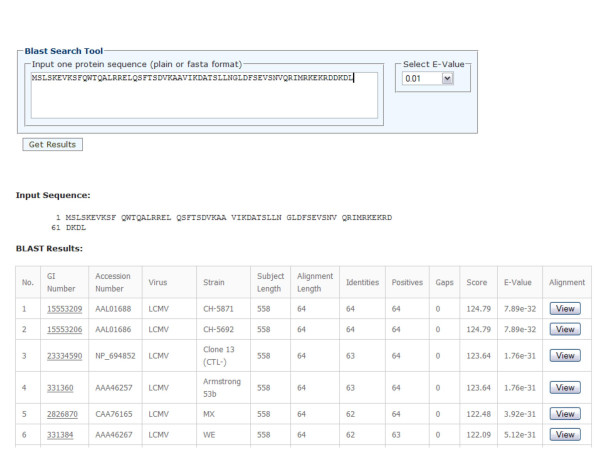
BLAST search interface.

The arenavirus protein sequence database has a convenient search interface allowing querying by virus, strain and protein names (Figure 2). All entries in the database are linked to the original NCBI records and cited PubMed references (if available). In addition, a tool utility is also provided that allows searching for arenavirus sequences containing specific peptides or epitope sequences (Figure 3). For example, this would allow researchers to quickly determine whether known epitopes are expressed by various arenavirus strains and species. This information could therefore be used to develop arenaviral epitope-based diagnostics and/or vaccine constructs. A BLAST search was also implemented allowing users to search for similar sequences contained within the database (Figure 4).

### Arenavirus protein sequence variability analysis

Using the sequences in this database, we further investigated the arenaviral protein sequence conservancy/variability. Our goal was to identify conserved or variable regions that could be targeted for development of a universal arenaviral vaccine or diagnostics, respectively. To do this, we performed multiple sequence alignments and entropy analyses between different strains of a virus and between different viruses.

Multiple sequence alignments were performed using CLUSTAL W program [21] using default parameters. To estimate the diversity a multiple protein sequence alignment, Shannon entropy (H) was calculated using equation 1 [22]:

H=−∑i=1MPilog⁡2Pi     (1)
 MathType@MTEF@5@5@+=feaafiart1ev1aaatCvAUfKttLearuWrP9MDH5MBPbIqV92AaeXatLxBI9gBaebbnrfifHhDYfgasaacH8akY=wiFfYdH8Gipec8Eeeu0xXdbba9frFj0=OqFfea0dXdd9vqai=hGuQ8kuc9pgc9s8qqaq=dirpe0xb9q8qiLsFr0=vr0=vr0dc8meaabaqaciaacaGaaeqabaqabeGadaaakeaacqqGibascqGH9aqpcqGHsisldaaeWbqaaiabdcfaqnaaBaaaleaacqWGPbqAaeqaaOGagiiBaWMaei4Ba8Maei4zaC2aaSbaaSqaaiabikdaYaqabaGccqWGqbaucqWGPbqAaSqaaiabdMgaPjabg2da9iabigdaXaqaaiabd2eanbqdcqGHris5aOGaaCzcaiaaxMaadaqadaqaaiabigdaXaGaayjkaiaawMcaaaaa@44B7@

where *P*_*i *_is the fraction of residues of amino acid type *i*, and M is the number of amino acid types (20). H ranges from 0 (only one residue in present at that position) to 4.322 (all 20 residues are equally represented in that position). Typically, positions with H ≥ 2.0 are considered variable, whereas those with H ≤ 2 are consider conserved. Highly conserved positions are those with H ≤ 1.0 [23].

Shannon entropy analyses of protein sequences contained in our database indicated that arenavirus protein sequences are fairly conserved between different strains of the same virus, but less so between different viruses. This is consistent with the view that arenaviruses are relatively stable genetically with amino acid sequence homologies of 90–95% among different strains of the same virus species and of 44–63% for homologous proteins of different arenavirus species [24]. As a result, to develop a universal vaccine against different arenaviruses, a construct that contains epitopes conserved within each virus should be used. For the purpose of developing diagnostics, however, epitopes derived from non-conserved regions would be excellent candidates.

**Figure 5:**
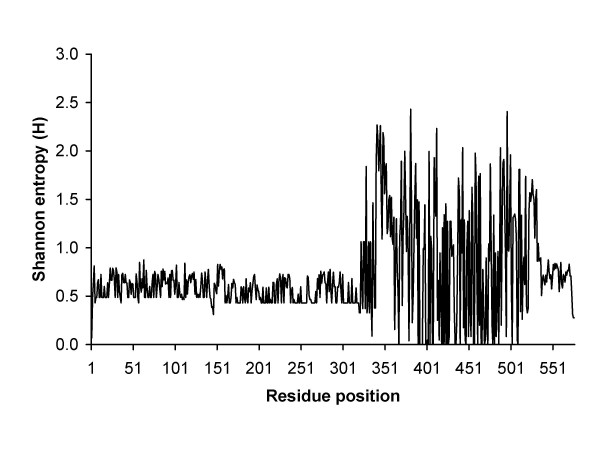
Inter-virus sequence diversity of NP protein.

**Figure 6:**
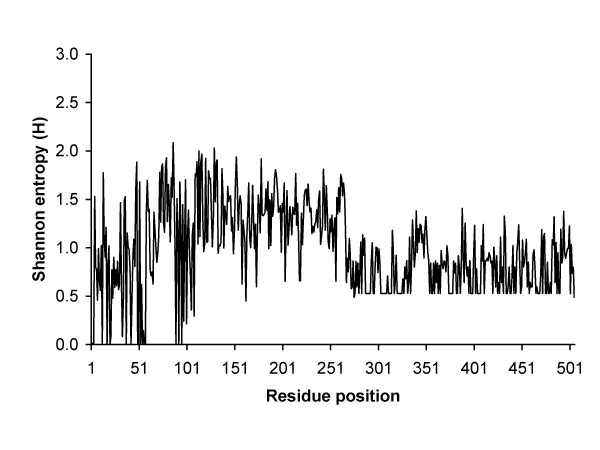
Inter-virus sequence diversity of GPC protein.

The most important arenaviral proteins are NP and GPC, and the NP proteins have been known as being the most conserved among arenaviruses. Our entropy analysis also revealed that the NP protein has a highly distinct inter-virus conserved region between residues 1–310 with average H ≈ 0.5 (Figure 5). Another distinct conserved region is in the GPC protein between residues 290–500 (Figure 6). As a result, epitopes derived from these regions have high probabilities to be cross-reactive between different arenaviruses. In contrast to long conserved regions observed in the NP and GPC proteins, the L and Z proteins have much shorter inter-virus conserved regions which could be related to the proteins' shared functional homology.

As curated in the Immune Epitope Database (IEDB) [25,26] and at the time of this analysis, epitopes derived from arenaviruses (mainly Lassa and LCMV), were exclusively from NP and GPC proteins. The majority of these epitopes were mouse MHC class I restricted and located in the conserved regions of NP and GPC proteins (data not shown). This indicates the identified T cell epitopes from NP and GPC proteins may cross-react among different arenavirus species. Nevertheless, whether these mouse MHC restricted epitopes would also be reactive in humans remains to be experimentally validated. It should be noted here that the lack of L and Z derived epitopes, as reported in IEDB, may imply that the curation is incomplete or more likely that no studies have yet been done to look for epitopes in these proteins.

## Conclusion

In conclusion, the database developed here, to our knowledge, is the only public resource that provides a non-redundant complete set of viral protein sequences for the seven highly pathogenic arenaviruses. These protein sequences can be used for epitope discovery studies, and their specific annotations are highly relevant for consideration in the complex task of developing diagnostics and/or vaccines. In another aspect, this database would also be a useful resource for scientists to investigate function-sequence conservation relationships among the arenaviruses.

## Availability and requirements

Project name: Arenavirus protein sequence database

Project homepage: 

Programming language: Java

Operating system: Fedora Linux

Other requirements: Apache Tomcat 5.5.12, MySQL 4.1, Firefox version 1.5 or higher

License: None

## List of abbreviations used

**AHF: **Argentine hemorrhagic fever

**BHF: **Bolivian hemorrhagic fever

**BrHF: **Brazillian hemorrhagic fever

**GPC: **Glycoprotein

**IEDB: **Immune Epitope Database and Analysis Resources

**LCMV: **Lymphocytic choriomeningitis virus

**LF: **Lassa fever

**MHC: **Major Histocompatibility complex

**NCBI: **National Center for Biotechnology Information

**NP: **Nucleoprotein

**VHF: **Venezuelan hemorrhagic fever

**WWA: **Whitewater Arroyo

## Competing interests

The author(s) declare that they have no competing interests.

## Authors' contributions

HHB developed the database and performed sequence variability analyses. JB manually annotated of the sequences. NF programmed the web interface. HHB, JB and AS wrote the manuscript. All authors participated in discussions, reviewed and approved the final manuscript version.
